# Current evidence and research gaps in menopause management in women with type 1 diabetes mellitus: a narrative review

**DOI:** 10.1530/EC-25-0486

**Published:** 2025-12-15

**Authors:** Aoife Courtney, Lisa Owens

**Affiliations:** Department of Endocrinology and Diabetes Mellitus, St James’s Hospital, Dublin, Ireland

**Keywords:** menopause, diabetes, hormone replacement therapy

## Abstract

The menopause transition represents a period of complex hormonal, metabolic, and psychosocial change that poses unique challenges for women living with type 1 diabetes mellitus (T1DM). Despite an expanding population of midlife women with T1DM, evidence to guide optimal menopause management remains limited. This narrative review synthesises current clinical and mechanistic evidence on the impact of menopause in women with T1DM. A literature search was conducted in MEDLINE (PubMed), the Cochrane Library, and professional society guidelines between January and May 2025. Across studies, women with T1DM appear to experience an earlier onset of menopause and an increased risk of osteoporosis, cardiovascular disease, psychological distress, metabolic deterioration and sexual dysfunction compared with women without diabetes. Oestrogen deficiency may exacerbate insulin resistance, dyslipidaemia, and vascular dysfunction, while glycaemic variability and altered insulin requirements are frequently reported during the menopause transition. Evidence regarding the safety and efficacy of hormone replacement therapy (HRT) in this group is sparse. In the absence of disease-specific data, clinicians should adopt an individualised approach – screening proactively for menopausal symptoms, bone loss, and cardiovascular risk, tailoring HRT decisions based on individualised risk profiles, and recommending transdermal oestradiol when HRT is used. This review highlights the urgent need for dedicated research, evidence-based guidelines, and integrated clinical pathways to optimise menopause management and long-term outcomes for women living with T1DM.

## Introduction

Diabetes mellitus affects approximately 10% of women in Western populations, of whom less than 10% have type 1 diabetes mellitus (T1DM) (1). As advances in diabetes care continue to improve life expectancy, there is a growing need to better understand the unique challenges women with diabetes face during the menopausal transition. Reflecting this, a UK–Ireland priority-setting partnership ranked the impact of menopause on glycaemia and the optimal management of this transition as the third highest research priority in T1DM (2).

Despite this recognised importance, the relationship between menopause and T1DM, and its broader systemic consequences, remains poorly studied. A 2013 Cochrane review evaluating hormone replacement therapy (HRT) in women with T1DM identified only one small, underpowered trial that also included women with type 2 diabetes mellitus (T2DM) (3). Similarly, the 2018 European Menopause and Andropause Society (EMAS) clinical guide on menopause and diabetes makes minimal reference to T1DM, and a more recent 2022 *Nature Reviews Endocrinology* article focuses primarily on T2DM (4, 5). This gap in evidence leaves women and healthcare providers with little guidance, which may contribute to suboptimal management of menopausal symptoms and related health risks in this cohort.

**Figure 1:**
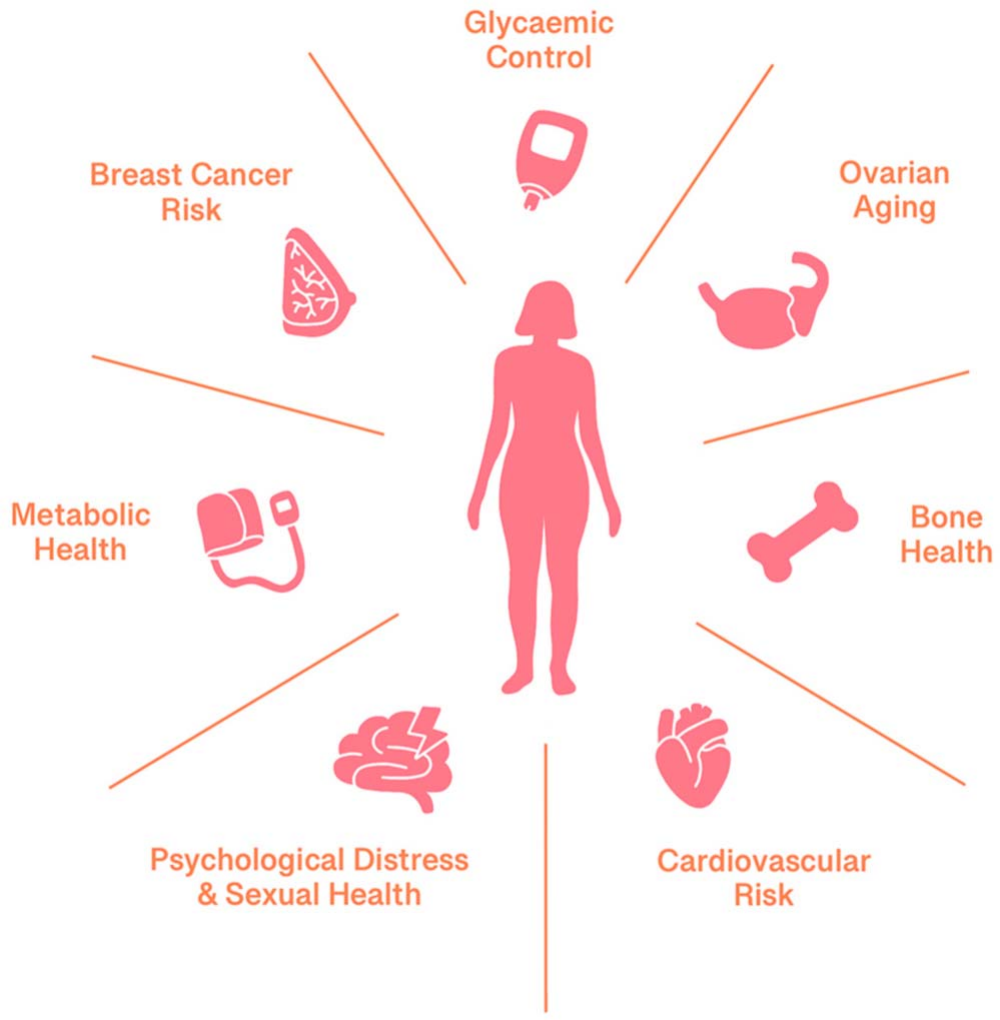
Overview of principal menopause-related health domains affected in women with type 1 diabetes mellitus.

This review explores the limited but evolving evidence on menopause in women with T1DM, including age of onset, changes in glycaemic control and insulin requirements, and the effects on bone, metabolic, and cardiovascular health, as well as breast cancer risk and psychological well-being. The potential role of menopausal hormone therapy (HRT) in managing these outcomes is also examined, and it highlights the areas where there is an urgent need for further research. The key menopause-related health domains examined in this review are illustrated in Fig. 1. Table 1 outlines practical recommendations for clinicians managing menopause in women with T1DM.

**Table 1:** Practical management considerations for women with type 1 diabetes mellitus during menopause.

Domain	Clinical issue	Suggested actions	Notes/evidence gaps
Menopausal symptoms	Potentially earlier onset; unique risk profile	Screen for menopausal symptoms in women living with T1DM, discuss lack of evidence, assist informed decision making. If commencing HRT preference for transdermal oestradiol use	Evidence on optimal HRT regimen is limited – clinical decision relies on extrapolation and individualised assessment
Glycaemic control	Potential fluctuation in insulin sensitivity during the menopause transition	Use CGM, smart pens, and hybrid closed-loop systems where available to track glycaemic variability and adapt insulin dosing	No RCTs in T1DM; extrapolated from general diabetes and menopause studies
Bone health	Accelerated BMD loss and fracture risk	Assess fracture risk in postmenopausal women >40 years using FRAX + BMD; consider adding trabecular bone score where available	No T1DM-specific fracture risk tool
		Ensure calcium/vitamin D sufficiency	No RCTs of osteoporosis therapy in T1DM
Metabolic health	Dyslipidaemia, adiposity, hypertension	Monitor lipids and BP regularly. Manage hypertension and dyslipidaemia per general guidelines; statin and antihypertensive therapy as indicated. Promote healthy lifestyle	Lipid response to HRT attenuated in T1DM; few data on BP control; no data on changes in body composition
Cardiovascular risk	High baseline risk, amplified post-menopause	Comprehensive CVD risk assessment: optimise BP, lipids, smoking cessation, physical activity. If considering HRT, prefer transdermal oestradiol ± micronised progesterone	No RCTs in T1DM. Women with longstanding T1DM generally considered high CVD risk
Breast cancer risk	Potentially altered risk profile in T1DM	Use standard breast cancer screening (e.g. mammography per local guidance). Discuss HRT risks and benefits individually; consider progestogen type if prescribing combined therapy	Conflicting epidemiology; no T1DM-specific HRT safety data
Sexual health	FSD, GSM	Screen routinely for FSD and GSM (consider FSFI). Offer vaginal oestrogen for GSM; lifestyle + glycaemic optimization; consider trial of transdermal testosterone for persistent low libido despite oestrogen	No T1DM-specific treatment studies; testosterone untested in this population
Psychological health	Depression, anxiety, diabetes distress	Screen with validated tools. Address overlap of vasomotor and hypoglycaemia symptoms. Provide access to psychological support and peer networks	Qualitative evidence only; no interventional trials in T1DM

Abbreviations: CGM, continuous glucose monitoring; RCT, randomised controlled trial; T1DM, type 1 diabetes mellitus; BMD, bone mineral density; BP, blood pressure; HRT, hormone replacement therapy; CVD, cardiovascular disease; FSD, female sexual dysfunction; GSM, genitourinary syndrome of menopause; FSFI, female sexual function index.

## Materials and methods

This narrative review synthesises clinical and mechanistic evidence on menopause in women with T1DM. Literature searches were conducted between January and May 2025 in the Cochrane Library and MEDLINE (PubMed). Search strategies combined terms such as ‘menopause’, ‘menopausal transition’, ‘type 1 diabetes’, ‘hormone replacement therapy’, and ‘menopausal hormone therapy’, together with controlled vocabulary relevant to each clinical domain. No study type filters were applied, although only articles published in English were included. To capture clinical guidance and consensus statements, we searched professional society websites (American Diabetes Association, British Menopause Society, Endocrine Society, European Menopause and Andropause Society, and European Society of Cardiology). Reference lists of included papers and relevant reviews were also screened. Evidence was synthesised thematically by clinical domain, prioritising the highest level of available evidence. A summary of the available evidence on the effects of type 1 diabetes mellitus on menopause-related outcomes across clinical domains is presented in Table 2.

**Table 2:** Summary of the key evidence regarding the effects of type 1 diabetes mellitus on menopause-related outcomes.

Domain	Study details	Population	Comparator	Key findings	Comments and limitations
Age of menopause	Dorman (2001) (9)	Women with T1DM (*n* = 143)	Sisters and unrelated controls without diabetes (*n* = 346)	Lower mean age at menopause in women with T1DM (41.6 years) versus sisters (49.9 years) and healthy controls (48.0 years) (*P* = 0.05)	Observational, self-reported data
	USA				
	Observational cohort				
	Sjöberg (2011) (15)	Women with T1DM (*n* = 476)	Finnish general population data	Median age of onset of menopause in women with T1DM not lower than general population (52.5 versus 51 years)	Population level data limits ability to control for confounders
	Finland				
	Cross sectional cohort				
	Brand (2015) (12)	Women with diabetes (*n* = 5,999)	Controls without diabetes (*n* = 252,899)	Early onset diabetes associated with earlier menopause (HR = 1.44 for onset between 10 and 20 years; HR = 1.59 for onset <10 years)	No distinction between T1DM and T2DM, self-reported diabetes status
	Europe				
	Prospective cohort				
	Yarde (2015) (18)	Women with T1DM (*n* = 140)	Controls without diabetes (*n* = 5,426)	No difference in mean age of natural menopause between women with T1DM and controls (49.8 versus 49.8 years, 95% CI: −0.34, 1.01)	No data on microvascular complications; survival bias
	Netherlands				
	Cross sectional cohort				
	Yi (2021) (11)	Women with T1DM (*n* = 105)	Controls without diabetes (*n* = 178)	Women with pre-menarche onset T1DM younger at menopause (−2.0 years, *P* < 0.0001)	Variable ascertainment of menopausal status, younger age of included women with T1DM, selection biases
	USA				
	Prospective cohort				
	Mehra (2023) (10)	Women with T1DM (weighted *n* = 5,138)	Controls without diabetes (weighted *n* = 1,336,779)	Diagnosis of T1DM before 30 years’ experience earlier menopause (HR 1.55) compared to controls	Self-reported data
	Canada				
	Retrospective cohort				
Bone health	Vestergaard (2007) (34)	Men and women with T1DM		RR = 6.94 (95% CI: 3.245, 14.78) for hip fracture in T1DM; BMD Z-score decreased in the spine (mean ± SEM −0.22 ± 0.01) and hip (−0.37 ± 0.16) in T1D	Small number of included studies for T1DM
	Systematic review and meta-analysis				
	Khalil (2011) (32)	Women with diabetes (*n* = 117)	Controls without diabetes (*n* = 2,054)	Women with diabetes experienced more rapid hip BMD decline (B = −0.45 vs −0.11 g/cm^2^/year; *P* < 0.001) and higher fracture incidence (RR = 2.2, 95% CI: 1.26–3.85)	Self-reported data, small number of fracture events
	USA				
	Longitudinal cohort				
	Shah (2015) (35)	Men and women with T1DM (*n* = 27,300)		RR = 3.16 (95% CI: 1.51, 6.63) for any fracture in T1DM	Heterogeneity with regard to fracture assessment and classification of T1DM
	Systematic review and meta-analysis				
	Weber (2015) (36)	Men and women with T1DM (*n* = 30,394)	Controls without diabetes (*n* = 303,872)	Increased fracture risk across all age groups; in women, highest overall fracture risk at 40–45 years (HR 2.03; 95% CI: 1.73, 2.39) and peak hip fracture risk at 30–39 years (HR 5.63; 95% CI: 2.25, 14.11)	Observational design, potential misclassification of T1DM, limited BMD/falls data
	UK				
	Population based cohort				
	Thong (2021) (31)	Women with T1DM (*n* = 107) and T2DM (*n* = 333)	Controls without diabetes (*n* = 10,873)	T1DM associated with increased risk of fracture (OR 2.28; 95% CI: 1.53, 3.40)	Self-reported data
	Australia				
	Longitudinal cohort				
Breast cancer risk	Boyle (2012) (73)	Women with T1DM and T2DM (*n* of studies = 40)		T1DM not associated with increased breast cancer risk (SRR 1.00; 95% CI: 0.74, 1.35); increased risk for women with diabetes (SRR 1.27; 95% CI: 1.16, 1.39)	Heterogeneous studies
	Systematic review and meta-analysis				
	Sona (2018) (74)	Men and women with T1DM (*n* of studies = 15)		Decreased risk of breast cancer in T1DM (OR or RR, 0.91; 95% CI, 0.86–0.95)	Heterogeneous studies
	Systematic review and meta-analysis				
	Xiong (2023) (72)	Women with T1DM (*n* = 575) and T2DM (*n* = 7,891)	Controls without diabetes (*n* = 235,025)	Women with T1DM had increased risk of breast cancer compared with controls (aHR 1.52; 95% CI: 1.03, 2.23)	Majority of white women; survival bias
	UK				
	Population based cohort				
Cardiovascular health	Lind (2014) (58)	Men and women with T1DM (*n* = 33,915)	Matched controls from the general population (*n* = 169,249)	People with T1DM had higher cardiovascular mortality than controls (aHR 4.6; 95% CI: 3.47–6.10); among those with T1DM aged 50–64 years, women had a greater risk than men (HR 7.92 vs 3.17)	People with T2DM potentially included in controls
	Sweden				
	Registry based observational cohort				
	Huxley (2015) (57)	Men and women with T1DM (*n* of studies = 26)		Pooled analysis (*n* = 75,983; 2,166 events) showed cardiovascular mortality was higher in people with T1DM versus controls (SMR 11.3 in women vs 5.7 in men; pooled rSMR 1.86, 95% CI: 1.62–2.15)	Heterogeneous studies
	Systematic review and meta-analysis				
Sexual health	Zhang (2023) (87)	Women with T1DM (*n* = 2,151)		Pooled prevalence of FSD in T1DM 38.5% (95% CI: 32.1, 45.0%); OR 3.77 (95% CI: 2.24, 6.35) compared to controls; longer diabetes duration and depression predictors of FSD in T1DM; vulnerability during menopausal transition	Significant heterogeneity
	Systematic review and meta-analysis				
Psychological health	Roy (2012) (91)	Men and women with T1DM (*n* of studies = 20)		Higher prevalence of depression in people with T1DM compared with controls (12%, range 5.8–43.3 vs 3.2%, range 2.7–11.4%) and in women than men with T1DM	Variable diagnosis of depression and diabetes, many cross sectional studies
	Systematic review and meta-analysis				
	Mackay (2014) (96)	Menopausal women with T1DM (*n* = 10)		Absence of information regarding menopause for women with T1DM; menopausal symptoms mimicking hypoglycaemia symptoms; erratic glycaemia; heightened anxiety during menopause transition	Caucasian women only, small sample size, limited generalisability
	UK				
	Qualitative exploratory				
	Forde (2024) (97)	Women with T1DM (*n* = 184)		Lack of awareness of the impacts of T1DM on menopause; increased disease burden during menopausal transition; overlap of hypoglycaemia and menopausal symptoms; difficulties in communication with HCPs	Limited generalisability
	UK				
	PPI survey				

Abbreviations: T1DM, type 1 diabetes mellitus; T2DM, type 2 diabetes mellitus; HR, hazard ratio; RR, relative risk; BMD, bone mineral density; OR, odds ratio; SRR, standardised relative risk; aHR, adjusted hazard ratio; SMR, standardised mortality ratio; rSMR, ratio of the standardised mortality ratio; FSD, female sexual dysfunction; PPI, patient public involvement; HCPs, health care professionals.

## Physiology of menopause

Menopause is defined as the permanent cessation of menstruation resulting from the loss of ovarian follicular activity (5). Spontaneous menopause is diagnosed retrospectively after 12 consecutive months of amenorrhoea and typically occurs between the ages of 48 and 52, although substantial variability exists across populations (6). The menopausal transition is characterised by menstrual cycle irregularity and fluctuating oestrogen levels, culminating in the final menstrual period (6).

Several processes contribute to the decline in ovarian function, including hypothalamic and ovarian ageing, as well as genetic, environmental, systemic, and lifestyle factors (7). Hypothalamic–pituitary ageing results in diminished synchrony of gonadotrophin-releasing hormone (GnRH), follicle-stimulating hormone, and luteinising hormone release (7). Together with ovarian ageing, these central changes impair follicular maturation and ovulation, and reduce the production of ovarian hormones, including inhibin B, anti-Müllerian hormone (AMH), and oestradiol (7). In contrast, ovarian androgen production is relatively preserved, leading to a state of relative androgen excess (5).

These physiological changes underlie menopausal symptoms such as hot flushes, night sweats, sleep and mood disturbance, myalgia, and arthralgia (7). Urogenital atrophy due to oestrogen deficiency may result in vaginal dryness, dyspareunia, urinary symptoms, and recurrent urogenital infections (7). Oestrogen deficiency also leads to uncoupled bone resorption and formation processes, leading to an increased risk of osteoporosis and fracture (7). In addition, menopause is associated with adverse metabolic changes, including dyslipidaemia, insulin resistance, central and visceral adiposity, and endothelial dysfunction (5, 8).

Overall, menopause represents a complex endocrinological transition with systemic consequences, driven primarily by the loss of ovarian oestrogen production.

## Age of onset of menopause in women with T1DM

Emerging evidence suggests that women with T1DM may experience natural menopause at a younger age compared to women without diabetes (9, 10, 11, 12). An earlier menopause carries important clinical implications, including a reduction in reproductive lifespan and increased risks of cardiovascular disease, osteoporosis, and overall mortality (11). Despite these potential consequences, the evidence base remains limited and somewhat inconsistent.

One of the earliest studies in this area, the Familial Autoimmune and Diabetes (FAD) study, reported a 17% reduction in reproductive years among women with T1DM compared with their sisters and unrelated controls without diabetes (9). Supporting these findings, Soto *et al.* observed that women with T1DM had lower levels of inhibin B and an earlier decline in AMH, suggesting an accelerated loss of ovarian reserve (13).

Diabetes duration and age of onset may influence the timing of menopause. The combined analysis of the Pittsburgh Epidemiology of Diabetes Complications (EDC) study and the Study of Women’s Health Across the Nation (SWAN) examined 80 women with pre-menarche onset T1DM and found both a delayed menarche (by 0.5-year, *P* = 0.002) and an earlier natural menopause (by 2.0 years, *P* < 0.0001) compared to women without diabetes (11). This pattern was not observed in women who developed T1DM after menarche, although this subgroup was relatively small (*n* = 25).

The European Prospective Investigation into Cancer and Nutrition (EPIC) study also identified an association between earlier-onset diabetes (diagnosed before age 20) and earlier menopause, with a hazard ratio (HR) of 1.43 (95% CI: 1.02–2.01) for onset between ages 10 and 20, and 1.59 (95% CI: 1.03–2.43) for onset before age 10 (12). However, the study did not distinguish between T1DM and T2DM. Similarly, a recent Canadian longitudinal study including 11,436 women found that women with T1DM diagnosed before age 30 were significantly more likely to experience earlier menopause (HR 1.55; 95% CI: 1.05–2.28) than women without diabetes (10).

The development of microvascular complications may be associated with age of menopause. Yi *et al.* found that women who developed microalbuminuria before age 30 experienced menopause approximately 2 years earlier than those with normoalbuminuria (*β* ± SE = −2.06 ± 1.08, *P* = 0.06) (14). However, no association was observed among women who developed microalbuminuria after age 30, nor with other diabetes-related complications. Similarly, Sjoberg *et al.* found that age-adjusted menopause prevalence was significantly associated with end-stage renal disease and proliferative retinopathy (15).

Conversely, findings from the Diabetes Control and Complications Trial (DCCT) and its long-term follow-up, the Epidemiology of Diabetes Interventions and Complications (EDIC) study, did not support a relationship between glycaemic control or microvascular disease and age at menopause (16). In a secondary analysis of 240 women from these studies, menopausal age did not differ significantly between the intensive and conventional treatment arms. Interestingly, higher insulin doses were associated with a lower risk of early menopause, suggesting a possible ovarian trophic effect of insulin – though this may have been a chance finding (16). In contrast, data from the EDC study indicated that both higher average insulin doses and increased albumin excretion over time were associated with earlier menopause (17).

Not all studies support an association between T1DM and early menopause. The OVIDA study, which included 140 postmenopausal Caucasian women with T1DM, found no significant difference in menopausal age compared to controls without diabetes (18). However, this study lacked data on microvascular complications and may have been affected by survival bias, potentially under-representing women with poor glycaemic control or advanced complications. Likewise, a Finnish study of 476 women with childhood-onset T1DM found no difference in menopausal age compared to national population estimates, though its reliance on population-level data limited the ability to control for confounders (15).

In summary, while many studies suggest that T1DM is associated with an earlier onset of natural menopause – particularly among those diagnosed at a young age or with microvascular complications – findings are not uniform. Discrepancies may reflect differences in study design, populations, and adjustment for confounders. Given the potential clinical implications, there is a clear need for large-scale, prospective studies to clarify this relationship and elucidate underlying mechanisms.

## Glucose control and insulin dosing in perimenopause

The effects of physiological fluctuations in sex hormones on glycaemic control during puberty and pregnancy in women with T1DM are well established, with oestrogen and progesterone positively associated with measures of insulin sensitivity (19, 20, 21). However, the impact of the menopause transition and HRT use on glycaemic control in women with T1DM has not been studied.

Menopause is associated with alterations in glucose homoeostasis that are independent of ageing (22, 23). Declining oestradiol (E2) levels during the transition contribute to reduced insulin sensitivity, changes in body composition, increased central and visceral adiposity, and a shift toward androgenicity (5). In animal models, oestrogen deficiency induced by ovariectomy results in increased insulin resistance independent of body composition changes, a process reversed with oestrogen replacement (24).

Human studies support a similar pattern. The Canadian Longitudinal Study on Aging identified menopause as an independent risk factor for impaired glucose tolerance (22). A 2017 meta-analysis reported higher fasting glucose and insulin levels among postmenopausal women (25). Secondary analysis of the PREDICT UK trial found that postmenopausal women without diabetes exhibited greater glycaemic variability and higher postprandial glucose concentrations compared with pre- and perimenopausal counterparts (26). However, evidence specifically relating to women with T1DM during this transition remains sparse.

In women without diabetes, HRT (primarily oestrogen) has anti-diabetic actions – it has been shown to improve insulin resistance and fasting glucose (23, 27). A meta-analysis of 107 trials found that HRT reduced fasting glucose by 2.5% (95% CI: (1.5, 3.5)) and fasting insulin by 9.3% (95% CI: (4.9–13.7)) resulting in an estimated 30% reduction in the risk of developing diabetes (27). Proposed mechanisms include oestrogen-mediated effects on skeletal muscle and hepatic gluconeogenesis (23). In addition, it has been proposed that oestrogens improve beta cell survival and insulin secretion as assessed by C-peptide measurement (23). The magnitude and nature of these effects vary by oestrogen formulation, dose and route of administration, and the addition of progestogens (27).

Despite encouraging data in the general population, studies examining the impact of HRT on glycaemic control in women with T1DM are scarce. A 2006 meta-analysis suggested that HRT improves fasting glucose and insulin resistance in women with diabetes, but did not distinguish between T1DM and T2DM, nor did it stratify results by HbA1c, diabetes duration, or HRT formulation (27).

A 2013 systematic review identified only one relevant trial: a randomised study by Scott *et al.* that included 150 postmenopausal women (56 with T1DM) receiving either 17β-oestradiol with norethisterone acetate or placebo (28). The study found no significant effect on glycaemic control as measured by serum fructosamine. However, the trial was limited by a high dropout rate, lack of HbA1c data, and incomplete reporting of treatment regimens.

A more recent 2023 meta-analysis assessing the impact of HRT on glycaemic control in diabetes included 1,412 participants, 4% of whom had T1DM (29). While HRT reduced HbA1c in women with T2DM (mean difference −0.56% (95% CI: −0.80, −0.31)), conclusions could not be drawn for women with T1DM due to the small sample size. In addition, the study was underpowered to assess the effects of HRT on glucose regulation, postprandial glucose, or glucose-lowering medication use.

Thus, the impact of HRT on glycaemic outcomes in women with T1DM remains poorly understood, with existing studies limited by small sample sizes, inconsistent glycaemic metrics, and a lack of standardised treatment protocols. Future research must be adequately powered to assess the effects of various HRT regimens on glycaemic control, insulin sensitivity, and diabetes management in this population.

Advances in diabetes technology provide an opportunity to address this knowledge gap. Data from continuous glucose monitors, insulin smart pens, and hybrid closed-loop systems allow precise tracking of glucose fluctuations, insulin requirements, and glycaemic variability (30). Large-scale, prospective studies leveraging these technologies could generate robust evidence to guide clinical decision-making, ensuring optimal menopause management for women with T1DM. Investment in such research is critical to inform evidence-based clinical practice and improve long-term health outcomes for this population.

## Bone health

Bone health during menopause is an important consideration. Menopause-related oestrogen decline accelerates bone mineral density (BMD) loss and increases fracture risk (31, 32, 33). T1DM independently contributes to skeletal fragility: a 2007 meta-analysis reported BMD reductions of 22% at the spine and 37% at the hip compared to age- and sex-matched controls (34). A 2015 meta-analysis found that individuals with T1DM have a 3.16-fold increased risk of any fracture (95% CI: 1.51–6.63, *P* = 0.002) compared to people without diabetes (35). An increased fracture risk has been observed across all age groups (36).

The mechanisms contributing to bone fragility in T1DM are likely multifactorial. Key factors include insulin and insulin-like growth factor-1 (IGF-1) deficiency, chronic hyperglycaemia, accumulation of advanced glycation end products, increased marrow adiposity, and inflammation (37, 38). Fracture risk also correlates with diabetes-specific variables such as duration of disease, glycaemic control (HbA1c), and the presence of microvascular or cardiovascular complications (37).

While the skeletal effects of menopause and T1DM have been studied independently, their combined impact is less well understood. Animal studies suggest a synergistic detrimental effect: ovariectomised mice with experimentally induced T1DM showed significantly greater trabecular bone loss than those with either condition alone (39). This bone loss was associated with reduced osteoblast activity, increased marrow adiposity, and elevated tumour necrosis factor-alpha expression, suggesting that oestrogen deficiency and T1DM together amplify bone inflammation and impair bone formation (39).

Observational data support a compounded risk in humans. Women with T1DM experience accelerated BMD loss and elevated fracture rates during the menopause transition (31, 32, 36, 40). In a cohort study by Weber *et al.*, fracture incidence was highest in women with T1DM aged 40–49 years (HR 2.03 (95% CI: 1.73–2.39)), with peak hip fracture risk occurring even earlier, between ages 30 and 39 (HR 5.63 (95% CI: 2.25–14.11)) (36). These findings highlight the role of premature bone loss in T1DM and suggest that menopause may exacerbate skeletal vulnerability.

Further evidence from a large cohort study evaluating menopause and BMD found that women with diabetes experienced more rapid hip BMD decline (*B* = −0.45 vs −0.11 g/cm^2^/year; *P* < 0.001), earlier menopause onset by approximately 3 years (*P* = 0.002), and a higher fracture incidence (RR = 2.2, 95% CI: 1.26–3.85) than women without diabetes (32). Importantly, adjusting for menopausal status attenuated the excess fracture risk, suggesting that earlier menopause may be a key mediator. However, this study did not differentiate between T1DM and T2DM.

Similarly, prospective data from the Australian Longitudinal Study on Women’s Health, including 107 women with T1DM, found a higher risk of falls and fall-related injuries, shortened reproductive lifespan, and increased fracture risk (OR = 2.28, 95% CI: 1.53–3.40) compared to controls (31). Adjustment for falls and reproductive lifespan modestly reduced the estimated fracture risk by 10 and 4%, respectively.

Despite the clear impact of both T1DM and menopause on bone health, optimal strategies for osteoporosis prevention and fracture risk reduction in this population remain uncertain. The American Diabetes Association (ADA) recommend considering BMD assessment in people with T1DM over the age of 50 years, however, this is not specific to postmenopausal women (41). FRAX algorithm can be used to estimate 10-year probability of major osteoporotic fracture in people over the age of 40 years (38). T1DM status can be incorporated as a secondary cause of osteoporosis and increase fracture risk only when BMD is not included. Trabecular bone score (TBS), derived from DXA imaging, increases predicted fracture probability in people with T1DM similar to people without diabetes (38). Therefore, we would recommend fracture risk evaluation in all postmenopausal women with T1DM using FRAX in those >40 years of age, ideally with BMD and DXA-derived TBS.

While HRT reduces fracture risk in the general population, its benefit in T1DM is unclear (42). The Nurses’ Health Study found that HRT use did not significantly reduce hip fracture risk in women with T1DM (40). Similarly, the Australian Longitudinal Study on Women’s Health found that HRT modestly attenuated the association between T1DM and osteoporosis, but this effect was lost when reproductive lifespan was included in the model (31). In addition, in this study HRT use was independently associated with an increased risk of osteoporosis (OR 1.49), possibly reflecting selection bias, where women with low bone mass may be more likely to receive HRT (31).

There are currently no randomised controlled trials (RCTs) specifically evaluating the safety and efficacy of osteoporosis pharmacotherapy in women with T1DM. A systematic review found similar improvements in BMD and reductions in vertebral (alendronate, raloxifene) and non-vertebral (teriparatide) fracture risk in patients with and without diabetes (43). However, most included participants had T2DM, and many results were based on subgroup or post hoc analyses. Subgroup analysis of the FREEDOM and FREEDOM extension trials indicated that denosumab significantly increased BMD and decreased vertebral fracture risk in postmenopausal women with osteoporosis and diabetes, but did not distinguish between T1DM and T2DM (44).

Maintaining adequate calcium and vitamin D levels within age-specific recommendations may be particularly relevant for people with T1DM. In animal models, where streptozotocin (STZ) is used to induce diabetes, STZ-treated rodents experienced loss of trabecular bone, higher urinary calcium excretion, and altered vitamin D metabolic enzyme expression compared with controls (45).

In summary, both menopause and T1DM are major contributors to impaired bone health, and their combination appears to exert synergistic effects on bone loss and fracture risk. However, significant knowledge gaps remain regarding the underlying pathophysiology and optimal strategies for osteoporosis management in this population. Further research – particularly involving prospective studies and RCTs – is needed to clarify the roles of HRT and pharmacological therapies in preventing fractures and preserving bone health in women with T1DM.

## Metabolic health

The menopause transition is associated with adverse metabolic changes, including increases in visceral adiposity, insulin resistance, dyslipidaemia, and rising blood pressure (8). These alterations contribute to heightened cardiovascular risk in all women, but may be particularly consequential for those with T1DM, who already face an elevated baseline risk (5, 8).

Declining oestrogen levels during menopause are associated with unfavourable shifts in body composition, notably increased visceral and abdominal subcutaneous adiposity and reduced lean mass (46). Visceral adiposity plays a central role in metabolic dysfunction through the production of inflammatory cytokines, free fatty acids, and reactive oxygen species, ultimately promoting insulin resistance, and is an independent predictor of cardiovascular disease and mortality (8, 47).

In T1DM, insulin resistance has been independently associated with asymptomatic coronary artery calcification (CAC), even after adjustment for traditional cardiovascular risk factors (48). One study found that while men with T1DM initially had a higher CAC risk, this sex difference disappeared after adjusting for central adiposity – suggesting that fat distribution may partly explain the greater CAC burden observed in women with T1DM (48).

HRT improves body composition in postmenopausal women in the general population by reducing fat mass and preserving lean mass, effects largely attributed to oestrogen (49). However, no studies have specifically assessed these effects in women with T1DM.

Menopause is also associated with adverse changes in lipid profiles (8). The SWAN study reported significant increases in total cholesterol, low-density lipoprotein (LDL) cholesterol, lipoprotein(a), and triglycerides during the late perimenopausal and early postmenopausal periods (50). The risk of elevated LDL cholesterol was approximately doubled in postmenopausal versus premenopausal women (50). Data on high-density lipoprotein (HDL) changes are inconsistent (50, 51).

Oestrogen therapy improves lipid profiles in the general population, lowering total and LDL cholesterol while raising HDL cholesterol, with oral regimens having a more pronounced impact than transdermal preparations (27, 52, 53). Among progestogens, micronised progesterone and dydrogesterone appear lipid-neutral, while medroxyprogesterone acetate (MPA) and levonorgestrel may attenuate oestrogen’s beneficial effects (27, 53).

In women with T1DM, the lipid response to HRT appears blunted. Data from the Third National Health and Nutrition Examination Survey (NHANES III) showed that HRT users with diabetes had lower total and non-HDL cholesterol than non-users (225 vs 241 mg/dL and 169 vs 188 mg/dL, respectively), but no improvement in HDL cholesterol (54). Similarly, Robinson *et al.* found a diminished HDL response and exaggerated triglyceride rise in women with diabetes (55). In a small study of postmenopausal women with T1DM and T2DM using Kliofem® (17β-oestradiol/norethisterone acetate), no significant changes in lipid profiles were observed compared to non-users (28).

Menopause is also associated with rising systolic blood pressure and increased risk of hypertension (52). These changes may reflect both age-related vascular stiffening and the loss of oestrogen-mediated vasodilation (8, 52). A 2020 meta-analysis found that early menopause (<45 years) is associated with increased risk of arterial hypertension, a finding especially relevant for women with T1DM, who may experience earlier menopause (56). In the general population, HRT may modestly lower blood pressure, particularly with conjugated oestrogens, though effects vary with formulation and route of administration (27, 52).

Hypertension is a common comorbidity in T1DM and a major modifiable risk factor for both microvascular and macrovascular complications. However, limited evidence exists on how the menopause transition influences blood pressure control in women with T1DM. One small study found no significant change in blood pressure among postmenopausal women with T1DM using Kliofem® (28).

Taken together, menopause is a period of heightened metabolic vulnerability for women with T1DM. Despite this, longitudinal data on the trajectory and management of metabolic changes during menopause in this population remain scarce. Furthermore, the metabolic effects of HRT – well-established in the general population – are poorly understood in women with T1DM, underscoring the need for dedicated research to guide optimal care.

## Cardiovascular risk

T1DM is an established risk factor for cardiovascular disease (CVD), with risk further amplified for women following menopause (5). The interplay between T1DM, oestrogen deficiency, and traditional CVD risk factors presents unique challenges in this population. Understanding the cardiovascular implications of both menopause and HRT use in women with T1DM is critical for informed risk stratification and therapeutic decision making.

CVD remains a leading cause of mortality among people with T1DM (5). Notably, women with T1DM experience an excess risk of death from cardiovascular disease compared with men. A 2015 systematic review and meta-analysis reported an 86% increased risk of cardiovascular mortality in women with T1DM compared with men with T1DM (standardised mortality ratio 1.86; 95% CI: 1.62, 2.15) (57). Data from the Swedish National Diabetes Registry further highlight this disparity (58). Among 33,915 people with T1DM, women aged 50–64 years had a significantly higher HR for cardiovascular death (HR 7.92 (95% CI: 6.22, 10.08)) compared with men (HR 3.17 (95% CI: 2.70, 3.72)), potentially reflecting the impact of the menopause transition (58).

As previously discussed, menopause is associated with adverse cardiometabolic changes, largely driven by the decline in endogenous oestrogen, which normally exerts vasodilatory, anti-inflammatory, and lipid-modulating effects (8). Although it remains debated whether menopause independently increases cardiovascular events beyond chronological ageing, postmenopausal women with diabetes have consistently been shown to have a two- to three-fold increase in cardiovascular mortality compared to those without diabetes (59).

Early menopause (<45 years) is also associated with a significantly increased risk of coronary heart disease (CHD) (RR 1.5; 95% CI: 1.28–1.78) and fatal coronary events (RR 1.23; 95% CI: 0.98–1.53) (60). This may be especially relevant if women with T1DM experience earlier menopause, as some studies suggest.

Despite these clear risks, there are no randomised controlled trials evaluating the cardiovascular effects of HRT specifically in women with T1DM. In the general population, observational studies have linked HRT use with a reduced risk of CVD (61). Early results from the Women’s Health Initiative (WHI) RCT reported an increased risk of CHD in women receiving combined conjugated equine oestrogen (CEE) and medroxyprogesterone acetate (MPA) therapy compared with placebo (62). Subsequent long-term follow-up data, however, found no overall increase in CHD or cardiovascular mortality with HRT (63, 64).

Route of administration and timing of HRT initiation appear important. A Danish cohort study found no increased myocardial infarction risk overall and suggested a lower risk among women using transdermal oestrogen-only therapy compared to oral preparations (65). A 2015 Cochrane review reported a significant reduction in CHD events when HRT was initiated within 10 years of menopause onset (RR 0.52, 95% CI: 0.29–0.96), while the ELITE RCT showed that HRT slowed atherosclerosis progression when started early but not when delayed ≥10 years post menopause (66, 67). The Heart and Estrogen/Progestin Replacement Study (HERS) studied CEE and MPA and reported an increased risk of coronary events in the first year of use in women with pre-existing CVD, but a reduction at years 4 and 5 of use (68). The initial increase with HRT has been questioned because of an apparent initial decrease in cardiovascular events in the placebo arm. Nonetheless, caution is advised when considering HRT in women at high cardiovascular risk.

Some clinical guidelines generally advise against systemic HRT in women with high cardiovascular risk, recommending local (vaginal) oestrogen for symptom relief where necessary (6, 8, 69). For women at moderate cardiovascular risk, transdermal oestrogen – alone or with micronised progesterone – may be considered on an individualised basis (6, 69). In contrast, recent guidance from the British Menopause Society suggests that systemic HRT may be considered even in women with established CVD, provided that hormone type, route, and co-therapies such as statins are appropriately selected (70).

In women with T1DM, guidance on HRT use remains uncertain due to a lack of disease-specific evidence. RCTs and longitudinal studies are required to assess the impact of HRT on cardiovascular risk in this population. The 2023 EMAS guideline on menopause in diabetes provides recommendations for HRT use in T2DM only (4). A pooled analysis of women with prediabetes or T2DM demonstrated that HRT use was associated with a reduced risk of stroke, CHD, and atherosclerotic cardiovascular disease in white women (71). However, women with long-standing T1DM are typically considered to have high cardiovascular risk. In the absence of robust, disease-specific evidence, HRT prescribing in T1DM should involve individualised risk assessment, optimisation of modifiable risk factors, and shared decision making. For those opting to use HRT, transdermal oestradiol should be recommended.

## Breast cancer risk

Women with T1DM may have a distinct breast cancer risk profile, shaped by metabolic, hormonal, and inflammatory pathways. While the association between diabetes and cancer is more established in T2DM, the evidence regarding breast cancer risk in T1DM remains limited and inconsistent (72). In addition, as women with T1DM increasingly live beyond the menopause transition, understanding how diabetes-related factors and the use of HRT may modify breast cancer risk is critical.

Epidemiological findings have been conflicting. A large UK population-based study reported a significantly increased risk of breast cancer in women with T1DM compared to those without diabetes (adjusted hazard ratio (aHR) 1.52, 95% CI: 1.03–2.23) (72). In contrast, a meta-analysis including 40 studies found an elevated summary relative risk (SRR) of breast cancer in women with diabetes overall (SRR 1.27, 95% CI: 1.16–2.39), but no association was observed for T1DM or diabetes in premenopausal women (73). Another meta-analysis focussing on T1DM reported a reduced risk of breast cancer in people with T1DM (RR 0.91, 95% CI: 0.86–0.95) (74). These discrepancies may reflect heterogeneity in study design, population characteristics, and varying adjustment for confounders such as body composition, reproductive history, and drug treatments.

Several mechanisms have been proposed to explain a potential link between T1DM and breast cancer. Chronic hyperglycaemia may promote tumourigenesis through increased oxidative stress, DNA damage, and downregulation of antioxidant expression (73, 75). While hyperglycaemia has been associated with increased cancer mortality in epidemiological studies, a 2010 meta-analysis found no cancer risk reduction with intensive glycaemic control in T2DM, casting doubt on a causal relationship (76).

Exogenous insulin has also been hypothesised to have mitogenic effects. In T1DM, supraphysiological systemic insulin concentrations are often required to achieve adequate portal insulin levels. This systemic insulin excess, along with the potential mitogenic effects of insulin and some insulin analogues, may promote tumour growth, possibly via stimulation of the insulin-like growth factor 1 (IGF-1) axis, a pathway implicated in the pathogenesis of several cancers (77). However, evidence linking insulin use with breast cancer remains conflicting (78, 79). The extent to which these mechanisms meaningfully contribute to breast cancer risk in women with T1DM remains uncertain.

The impact of HRT on breast cancer risk in women with type 1 diabetes (T1DM) is not well defined. In the general population, combined oestrogen-progestogen HRT has been associated with a modestly increased risk of breast cancer, which varies by progestogen type and treatment duration (80). Data from the E3N cohort study found that HRT regimens containing micronised progesterone or dydrogesterone were associated with lower breast cancer risk compared to other progestogens (81). In contrast, oestrogen-only HRT appears to have little or no effect on breast cancer risk (80, 82).

A major limitation in many breast cancer risk analyses is the failure to account for baseline individual risk (61). A recent reanalysis of the Collaborative Group on Hormonal Factors in Breast Cancer (CGHFBC) meta-analysis highlighted that the absolute breast cancer risk associated with HRT varies substantially depending on a woman’s background risk profile (82). Importantly, none of these studies stratified results by diabetes type, limiting their applicability to women with T1DM.

The association between T1DM and postmenopausal breast cancer risk remains unclear. Some data suggest a modestly increased risk, while others report a decreased or null association. Mechanistic hypotheses – including hyperglycaemia and exogenous insulin – are not conclusively established. Regarding HRT, current evidence is largely extrapolated from the general population and does not adequately address the unique context of T1DM. Disease-specific research is needed to guide safe and individualised menopause management in this population.

## Sexual health

Female sexual dysfunction (FSD) is a common complication of menopause, with a reported prevalence of 40%, and can significantly impair quality of life (83). Diminished libido during the menopause transition has been shown to be independent of chronological ageing and associated with menopausal symptoms such as sleep disturbance, vasomotor symptoms, and mood disorders (83, 84). Genitourinary symptoms secondary to menopause-related oestrogen deficiency (genitourinary syndrome of menopause (GSM)), including vaginal dryness and discomfort, recurrent urogenital infections, urinary frequency, and urgency, further contribute to FSD (7, 85).

A number of studies have demonstrated that women with T1DM experience higher rates of FSD compared to controls and women with T2DM (41, 86). Proposed mechanisms include vascular dysfunction, hyperglycaemia, neuropathy, genitourinary infections, and psychosocial factors (86). A 2023 systematic review and meta-analysis of 19 studies including 2,151 women with T1DM, reported a pooled FSD prevalence of 38.5% (95% CI, 32.1–45.0%) (87). Rates were lower in premenopausal women (34.7%) compared with mixed peri- and postmenopausal groups (42.1%), suggesting a particular vulnerability around and after menopause.

Despite this, sexual health remains under-discussed in diabetes care (86). The 2025 ADA’s standards of care now recommend screening for FSD in women with diabetes, and specifically assessing for GSM symptoms in postmenopausal women (41). While no screening tools are validated for women with T1DM, the female sexual function index (FSFI; score ≤26.55 indicating FSD) is commonly used (86). Practical frameworks to support discussions of FSD in women with T1DM have recently been published (86).

There are no specific treatment guidelines for FSD in postmenopausal women with T1DM. The ADA advise the diagnosis and treatment of menopausal symptoms with hormonal therapies in conjunction with lifestyle interventions, optimising glycaemic control, and addressing psychosocial factors (41). A Cochrane review in 2023 found that oestrogen monotherapy slightly improves sexual function in those with menopausal symptoms, in early menopause, and in unselected postmenopausal women, while the effect of combined oestrogen-progestogen therapy, synthetic steroids, and selective oestrogen receptor modulators (SERMs), was uncertain (88).

The British Menopause Society recommends a trial of testosterone treatment for persistent low sexual desire despite adequate oestrogen therapy, with or without progestogen, aiming to maintain serum testosterone within the normal female range (80). A 2019 systematic review and meta-analysis supports the efficacy of testosterone in improving sexual function, with transdermal administration preferred due to its neutral effect on lipid profiles (89). To date, no trials of testosterone therapy have been conducted in women with T1DM.

## Psychological health

Women with T1DM are at increased risk of psychological distress throughout their lives (90). Depression and anxiety are more prevalent among individuals with T1DM compared to the general population, with a disproportionate burden observed in women; a systematic review has reported a threefold increased risk of depression in this population, with the highest prevalence noted among women (91). Importantly, both depression and diabetes-specific distress are associated with poorer glycaemic control, adverse lipid profile, reduced quality of life, and increased mortality (90, 92).

Menopause itself is a recognised contributor to mood disturbances, particularly during the perimenopausal period (93). The Harvard Study of Moods and Cycles found that perimenopausal women were nearly twice as likely to experience new-onset depressive symptoms compared to age-matched premenopausal controls (OR 1.8, 95% CI: 1.0, 3.2) (94). This effect appears to be more pronounced in those with a prior history of depression (95). Oestrogen therapy has been shown to be effective in the management of depressive disorders in perimenopausal women, both with and without vasomotor symptoms, although this benefit does not extend to postmenopausal women (93).

Despite these findings, the impact of menopause and HRT on psychological well-being and diabetes-specific distress in women with T1DM remains poorly characterised. Qualitative studies have identified key areas of concern expressed by women with T1DM during the menopause transition. One prominent source of distress is the overlap between vasomotor and hypoglycaemia symptoms. Both a 2014 qualitative study and a recent UK Patient and Public Involvement survey highlighted how symptoms associated with menopause such as night sweats, palpitations, and anxiety closely mimic those of hypoglycaemic episodes, complicating symptom interpretation and heightening anxiety (96, 97). For many, this ambiguity led to more frequent glucose monitoring, disrupted sleep, and a persistent fear of undetected nocturnal hypoglycaemia (96, 97).

Both studies also underscored the psychological burden stemming from a lack of clear, evidence-based guidance on managing menopause in the context of T1DM (96, 97). Women reported feeling unprepared for the physiological changes of menopause and frustrated by the absence of tailored information regarding its impact on diabetes management (96, 97). This uncertainty diminished confidence in self-management and contributed to feelings of isolation (96, 97).

Unpredictable glycaemic patterns were another consistent theme (96, 97). Many women experienced significant shifts in insulin requirements and glucose levels, leading to emotional exhaustion, a sense of lost control of their diabetes, and concerns regarding the long-term health implications of erratic glycaemia (96, 97).

Finally, a recurring theme was the perception that healthcare professionals frequently minimised or dismissed women’s self-reported experiences (96, 97). Women described difficulties in initiating conversations with clinicians, who were often perceived as lacking training or an evidence base to address menopause-related concerns in the context of T1DM (96, 97). Participants expressed a desire for improved clinician education, a multidisciplinary approach, and access to peer support networks to bridge this gap (96).

Together, these findings point to an urgent need for a stronger evidence base, improved education and training for healthcare providers, integration of menopause care into routine diabetes services, and the development of peer support resources. The diabetes research steering group, organised by Diabetes UK, recently conducted a workshop aimed at outlining key research priorities in the area of menopause and diabetes. This workshop highlighted six key areas for future research, one of which was the need to understand the impact of menopause on the mental health of women with diabetes and to study tailored psychological interventions to support women during this transition (98). Further research is also needed to explore the intersection between diabetes distress and the psychological impact of menopause in women with T1DM.

## Conclusions

The intersection of menopause and T1DM presents a constellation of challenges that are not adequately addressed in current clinical practice or research. Despite broad agreement that menopause compounds cardiometabolic and skeletal risk in women with T1DM, several areas remain controversial. Evidence regarding the timing of natural menopause in T1DM is inconsistent, with some studies reporting earlier onset and others finding no difference compared with controls. Similarly, the cardiovascular effects of HRT remain debated; while observational data in the general population suggest cardioprotective benefits when initiated near menopause, this has not been confirmed in diabetes-specific cohorts. The relationship between T1DM and breast cancer risk also remains unclear, with conflicting findings across epidemiological studies. Finally, while oestrogen therapy appears to improve bone density and metabolic parameters in the general population, its benefits in T1DM have not been demonstrated in randomised trials. These controversies highlight the need for adequately powered, longitudinal studies to clarify mechanisms and guide clinical care.

Optimising menopause management in women with T1DM requires a multidisciplinary, individualised approach, with careful consideration of glycaemic control, cardiovascular risk stratification, and bone health preservation (summarised in Table 1). Clinicians must also be equipped to address the psychosocial burden of menopause in this population. There is a potential benefit of HRT on bone health, cardiovascular risk factors, and disease for women with T1DM. Until dedicated research, however, is conducted, evidence-based extrapolation from studies in the general population must be approached with caution.

There is an urgent need for clinical trials and observational studies that include women with T1DM to develop tailored guidelines and ensure equitable, evidence-informed care during this important life stage.

## Declaration of interest

The authors declare that they have no competing interests. Lisa Owens is a Senior Editor of *Endocrine Connections*. Lisa Owens was not involved in the review or editorial process for this paper, on which she is listed as an author.

## Funding

This work did not receive any specific grant from any funding agency in the public, commercial, or not-for-profit sector.

## Author contribution statement

Both authors contributed to the study conception and design. The first draft of the manuscript was written by Aoife Courtney, and both authors commented on previous versions of the manuscript. Both authors read and approved the final manuscript.
